# Controlling GRF4‐GIF1 expression for efficient, genotype‐independent transformation across wheat cultivars

**DOI:** 10.1111/tpj.70799

**Published:** 2026-03-17

**Authors:** Sadiye Hayta, Mark A. Smedley, Meltem Bayraktar, Macarena Forner, Anna Backhaus, Clare Lister, Martha Clarke, Cristobal Uauy, Simon Griffiths

**Affiliations:** ^1^ John Innes Centre Department of Crop Genetics, Norwich Research Park Norwich UK; ^2^ Kırşehir Ahi Evran University Faculty of Engineering and Architecture, Department of Genetics and Bioengineering Kırşehir Turkiye; ^3^ Leibniz Institute of Plant Genetics and Crop Plant Research (IPK) Seeland Germany

**Keywords:** wheat, crop transformation, genotype independence, *GRF4‐GIF*, *Agrobacterium*, gene editing

## Abstract

Wheat is a staple crop critical for global food security, and its continuous genetic improvement is essential to meet the demands of a growing population. Efficient, genotype‐independent transformation is a major bottleneck in wheat functional genomics and gene editing. The growth regulating factor (GRF)–GRF‐interacting factor (GIF) fusion technology enhances regeneration efficiency and broadens the range of transformable cultivars, but constitutive expression can reduce fertility and spikelet number. Here, we present an optimised *Agrobacterium*‐mediated wheat transformation protocol incorporating *GRF4‐GIF1*, tested across multiple tetraploid and hexaploid cultivars. Transformation efficiency was improved through adjustments in selection pressure, zeatin concentration, and promoter choice, with *GRF4‐GIF1* consistently enabling successful transformation across genotypes. Tissue‐specific promoters and heat‐inducible excision strategies effectively minimised pleiotropic effects, such as reduced fertility, while maintaining high transformation rates. This refined system provides a robust and versatile platform for gene function studies and gene editing, advancing genotype‐independent wheat transformation and supporting breeding efforts to improve crop productivity, resilience, and nutritional value.

## Significance Statement

Improving wheat is vital for food security, yet many cultivars resist genetic transformation, slowing gene discovery and editing. We introduce a reliable, genotype‐independent transformation system that controls pleiotropic effects, enabling functional studies and precise genome editing across diverse cultivars and accelerating breeding for higher yield, resilience, and improved nutrition.

## INTRODUCTION

Bread wheat (*Triticum aestivum* L.), also known as common wheat, is one of the “big three” cereal crops worldwide Shewry (2009), with its extensive cultivation range and vital role as the primary source of cereal‐based processed products. Wheat significantly contributes to global food security, providing approximately 20% of the world's caloric intake and 25% of daily protein consumption. Furthermore, wheat is an important source of essential minerals for human nutrition and contributes up to 20% of essential dietary minerals in the UK (Sigalas et al., 2024). Durum wheat (*Triticum turgidum* L. ssp. *durum*), known for its high protein content, golden colour, and firm texture, serves as an important food source in certain regions; its production accounts for approximately 7% of total global wheat production, and about 75% of worldwide durum wheat production is used by the pasta industry (Grosse‐Heilmann et al., 2024).

Improving wheat genetics to enhance its nutritional value, disease resistance, and resilience to climate change will have a profound impact on sustainable food production and global nutritional security. The advent of advanced technologies, including the development of multiple reference‐quality genome assemblies, has ushered in a new era for wheat research, equipping researchers and breeders with vital tools to enhance wheat and address future food security challenges (Walkowiak et al., 2020). These genome assemblies provide a foundation for functional gene discovery and breeding, facilitating the development of the next generation of modern wheat cultivars. The availability of these genomic sequences, together with new open‐access transformation protocols (Hayta et al., 2019), videos, and plasmid repositories, will enable the powerful application of molecular tools in future wheat research and breeding. Effective transgenic methodologies are now crucial for conducting gene functional studies, enhancing traits, and integrating precision breeding techniques.

Wheat transformation is highly genotype‐dependent and typically relies on a few cultivars, such as Fielder and Bobwhite, which exhibit a strong tissue culture response (Hayta et al., 2019). Despite the first successful reports of *Agrobacterium*‐mediated wheat transformation in the 1990s (Cheng et al., 1997), transformation efficiencies remained low, around 5%, for many years. An *in planta* method was reported in 2009 (Risacher et al., 2009), but it was not widely adopted. Later, Japan Tobacco Inc. demonstrated improved transformation efficiency in the model wheat genotype Fielder using a patented, licensed system called PureWheat, which requires specialist training and specific vectors, thereby limiting its accessibility (Ishida et al., 2015).

Our first wheat CRISPR study, on the *ZIP4*‐B2 (*Ph1* locus), a meiotic gene involved in crossover within wheat (Rey et al., 2018), led to the publication of our freely available, efficient, and reproducible transformation protocol in 2019, achieving transformation efficiencies averaging around 25% in Fielder, and also Kronos and Cadenza with lower efficiencies of 10% and 4%, respectively (Hayta et al., 2021). Kronos and Cadenza are important reference varieties, with extensive TILLING resources available for gene discovery and complementation (Krasileva et al., 2017). The key factors influencing wheat transformation include the cultivar used, the quality and health of the donor material, the developmental stage of the immature embryos, the handling of the material, and the media component composition; transformation efficiencies are drastically affected by multiple factors, with these narrow optimal windows (Hayta et al., 2021).

The genotype independence of transformation techniques further broadens the potential for genetic improvements, making wheat breeding more versatile and accessible. Debernardi et al. (2020) developed a growth regulator fusion technology involving growth regulating factor 4 (GRF4) and its cofactor GRF‐interacting factor 1 (GIF1). Overexpressing *GRF4‐GIF1* in wheat significantly boosts regeneration efficiency and broadens the range of transformable wheat genotypes. We tested this technology using our published wheat transformation method. Fielder plants transformed with the *GRF4–GIF1* chimera increased dramatically to 77.5% efficiency (Debernardi et al., 2020). This technology accelerates the transformation pipeline and enables high transformation efficiencies by overcoming narrow optimal windows, such as specific growing conditions and embryo developmental stages, which are critical for successful transformation in wheat.

Low transgene copy number *GRF4‐GIF1* transgenic plants exhibited normal development and fertility in Fielder; however, constitutive expression of other plant developmental regulators such as the maize *Baby boom* (*ZmBbm*), the maize *Wuschel2* (*ZmWus2*) (Johnson et al., 2023), and a Wuschel homologue *TaWox5* (Wang et al., 2022) can lead to negative pleiotropic effects, necessitating their removal from transgenic plants and limiting their practical application (Wang et al., 2020). In an effort to minimise the negative pleiotropic effects associated with developmental regulators, Lowe et al. (2016) demonstrated a strategy for successful plant regeneration using Cre recombinase, which excised morphogenic gene sequences flanked by loxP sites. This approach was critical in maize transformation, removing unwanted expression cassettes (*Bbm* and *Wus2*) linked to morphological abnormalities including reduced root elongation and ensuring normal plant development. Further advancements were made by Lowe et al. (2018) who identified the *ZmPLTP* (maize phospholipid transferase protein gene), a maize promoter with strong expression in leaves, embryos, and callus, but downregulated in roots, meristems, and reproductive tissues. This promoter was used to drive Bbm expression in maize, enabling efficient somatic embryo formation without the callus phase and rapid plant regeneration. The *ZmPLTP* allowed for uniform transformation and successful regeneration of maize plantlets. Co‐transformation has been widely used because it is a simple and clean technique, leaving no residual DNA sequences, such as inverted repeats and recombination sites, in transgenic plants from which the selectable marker gene has been eliminated with high frequency. Co‐transformation involves the simultaneous integration of a selectable marker gene and a gene of interest from different T‐DNAs, followed by their subsequent recombination and segregation in the progeny, provided the two genes are integrated into unlinked loci (Liu et al., 2020).

Debernardi et al. (2020) demonstrated that transgenic plants overexpressing the *GRF4‐GIF1* chimera under the control of the *ZmUbi* promoter maintained fertility and morphology. However, these plants exhibited a 23.9% reduction in grain number per spike and a 13.7% increase in grain weight, suggesting that *GRF4‐GIF1* can modulate physiological traits without drastically impairing plant development. In our study, high transgene copy numbers of *GRF4‐GIF1* were associated with fertility depending on the cultivar but also showed a modest positive effect on grain area and grain length. For effective gene function characterisation, it is critical to develop transgenic plants that exhibit desirable traits while minimising adverse effects linked to morphological gene overexpression. The *GRF4–GIF1* technology addresses genotype dependence and expands the applicability of transformation methods, including gene editing tools, across elite wheat cultivars. Moreover, the ability to segregate or remove *GRF4–GIF1* after transformation provides enhanced flexibility for downstream applications, such as functional genomics, gene editing, and precision breeding. Importantly, *GRF4–GIF1* technology, together with open protocols, further democratises gene editing by making these approaches increasingly accessible to research centres and wheat breeders lacking high‐end facilities.

Despite the efficiency gains offered by *GRF4–GIF1*, its constitutive expression can lead to pleiotropic effects, including reduced fertility, spikelet number, and altered plant architecture, which vary across genotypes. Promoter strength, temporal activity, and construct design, as well as media composition such as zeatin and hygromycin levels, can influence both transformation efficiency and phenotypic outcomes. Additionally, we propose strategies to limit the temporal activity of *GRF4–GIF1*, such as using a PLTP tissue‐specific promoter, excision of *GRF4–GIF1*, and co‐transformation with two *Agrobacterium* plasmids, one carrying the *GRF4–GIF1* expression cassette and the other carrying both the selectable marker and the gene of interest (GOI). These strategies were tested across multiple wheat cultivars to mitigate undesirable pleiotropic effects, such as reduced grain number. Together, these refinements enhance the accuracy and reliability of functional analyses for trait‐associated genes, enabling efficient wheat transformation while maintaining developmental stability.

## RESULTS AND DISCUSSION

### GRF transcription factors and physiological impact

GRF transcription factors are highly conserved across angiosperms, gymnosperms, and mosses, encoding proteins with conserved QLQ (Gln, Leu, and Gln) and WRC (Trp, Arg, and Cys) domains essential for protein–protein and protein–DNA interactions. In many plant lineages, GRFs are regulated by microRNA miR396, which downregulates GRF expression in mature tissues (Debernardi et al., 2014).

**Figure 1:**
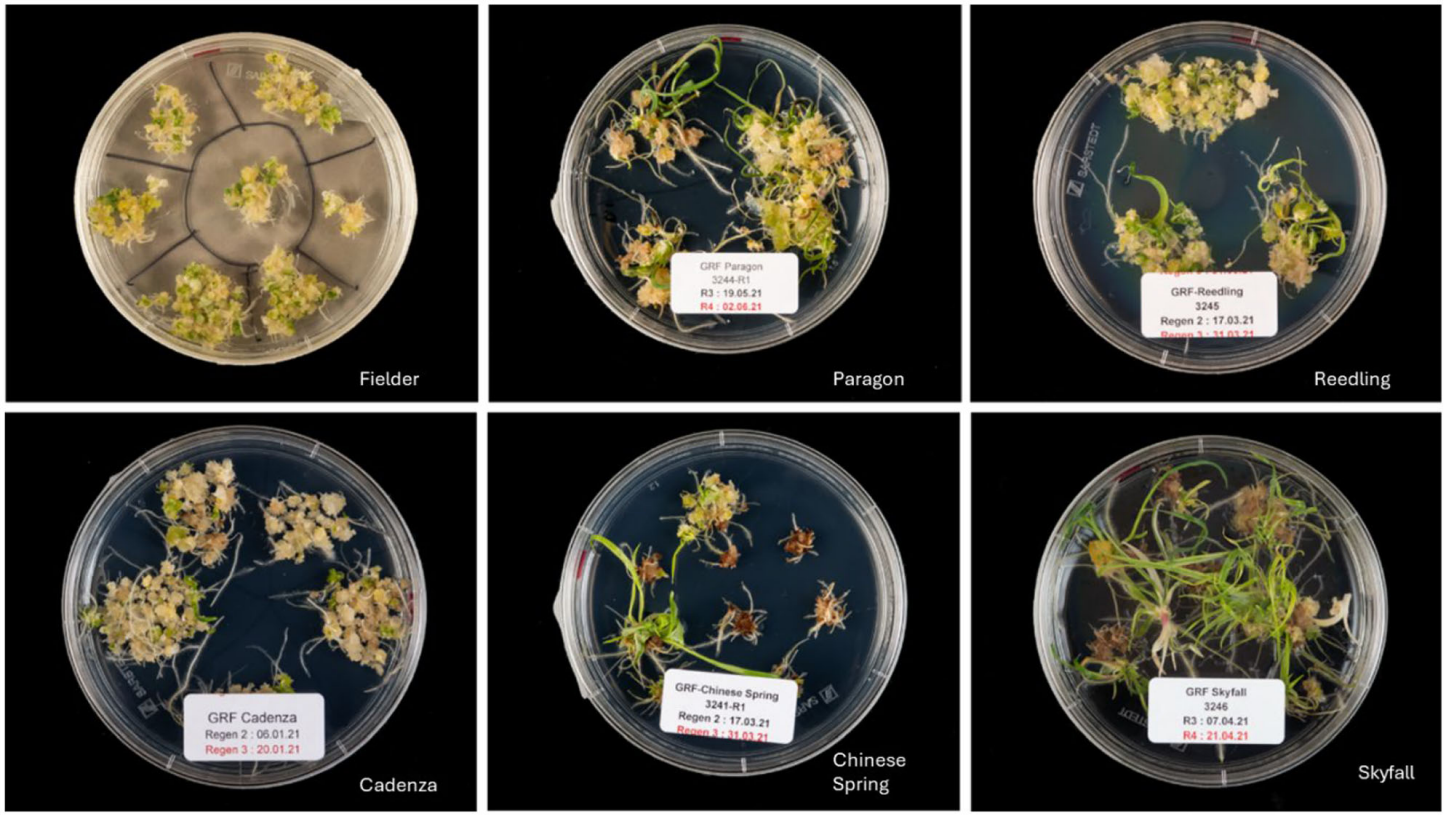
Regeneration of wheat calli using the *ZmUbi GRF4‐GIF1* construct across different cultivars. Representative regeneration responses are shown for the wheat cultivars Fielder, Reedling (Borlaug 100), Cadenza, Chinese Spring, and Skyfall, respectively.

We first tested the *ZmUbi GRF4‐GIF1* construct with nine hexaploid wheat cultivars (Table 1), including several that had previously been considered recalcitrant to transformation. A construct with GUS reporter driven with *OsUbi* promoter used as the control. For *GRF4‐GIF1* transformations, 70–100 immature embryos were used per cultivar, and 50–80 embryos were used for controls. Calli transformed with *ZmUbi::GRF4‐GIF1*, successfully produced somatic embryos and regenerated plantlets (Figure 1; Figure S1). using *ZmUbi::GRF4‐GIF1*, which successfully produced somatic embryos and regenerated plantlets (Figure 1; Figure S1). These results are consistent with Debernardi et al. (2020), who reported that the *GRF4‐GIF1* chimera promotes embryogenesis, shoot proliferation, or both in wheat, thereby improving transformation efficiency, particularly in recalcitrant cultivars.

**Table 1:** Hexaploid and tetraploid wheat cultivars used in transformation experiments, including each cultivar's classification, genome sequence availability, and whether it has been transformed previously

Genotype name	Type of wheat	Full genome sequence	Transformed previously
Fielder	US spring wheat	Yes	Yes
Cadenza	UK alternative wheat	Yes	Yes
Paragon	UK spring wheat	Yes	No
Reedling (Borlaug 100)	CIMMYT variety	Yes	No
Chinese Spring	Chinese spring wheat variety	Yes	No
Skyfall	Group 3 winter wheat	No	No
RGT Rashid	Group 3 winter wheat	No	No
Valoris	French winter wheat	No	No
Capelle Desprez	UK winter wheat	No	No
OmRabi5	ICARDA spring durum wheat	No	No
CIRNO‐C	CIMMYT spring durum wheat	No	No
Svevo	Spring durum wheat	Yes	No
Kronos	Spring durum wheat	Yes	Yes

In wheat, *in vitro* regeneration can proceed via two main pathways: somatic embryogenesis, in which a single somatic cell from embryos develops into complete plants, and organogenesis, where shoots or roots are initiated from callus or directly from explants. Somatic embryogenesis is considered the more fundamental mode of regeneration because it produces progeny with high genetic stability and reduced somaclonal variation. This pathway is particularly important in wheat, as it enables the transformation and regeneration of genotypes that are otherwise recalcitrant to *in vitro* culture (Kowalik et al., 2026).

In our system, transformation with *ZmUbi::GRF4‐GIF1* primarily promotes somatic embryogenesis, generating multiple embryos from calli delivered from immature embryo explants which subsequently develop shoots and fully regenerated plantlets. Enhancing embryogenic competence with *GRF4‐GIF1* is critical for cultivars that normally exhibit low regeneration capacity, as it increases the number of transformable cells and improves overall transformation efficiency.

Our construct carries both the hygromycin selectable marker and the *GRF4‐GIF1* chimera on the same T‐DNA. Therefore, the transgene copy number, as measured by Hyg integration, reflects the number of *GRF4‐GIF1* copies present in each transformed line. Data from mature plants indicated that the degree of fertility loss associated with *GRF4‐GIF1* expression varied among cultivars (Figure S2). Lines with higher transgene copy numbers exhibited a slightly increased proportion of sterile plants, although the severity of this effect depended on the specific cultivar. These results demonstrate a clear relationship between the number of integrated T‐DNA copies and developmental outcomes, providing a mechanistic explanation for observed differences in fertility across genotypes.

Among the cultivars tested, Kronos had the lowest seed set, indicating the greatest sensitivity to *GRF4‐GIF1* whereas Paragon and Cadenza were less affected (Figure S1). *GRF4‐GIF1* had a slightly positive effect on grain area and grain length across all cultivars, consistent with findings by Debernardi et al. (2020) who reported a 23.9% reduction in grain number per spike and a 13.7% increase in grain weight. A significant increase in the number of spikes was observed in Fielder (26.1 spikes per plant) compared with the control (11.8 spikes per plant).

### Transformation efficiency and phenotypic effects in hexaploid wheat

We first optimised wheat transformation using pGGG constructs containing the *PvUbi* promoter driving the hygromicin selection gene with the CAT1 intron, and the rice ubiquitin promoter driving a GUS reporter gene containing two introns in Fielder, Reedling (Borlaug 100), Cadenza, Chinese Spring, and Skyfall. Using the same selection system, we tested a construct in which the *ZmUbi* promoter drives *GRF4‐GIF1* to assess its effect on wheat transformation efficiency. After the initial callus induction stage, cultures grown under light conditions initiated a significantly greater amount of shoot formation (Figure 1) compared with those transformed with the control construct. Both shoot and root development appeared normal, producing an average of 3.2 plantlets per embryo. All regenerated plantlets tested positive for the transgene.

**Figure 2:**
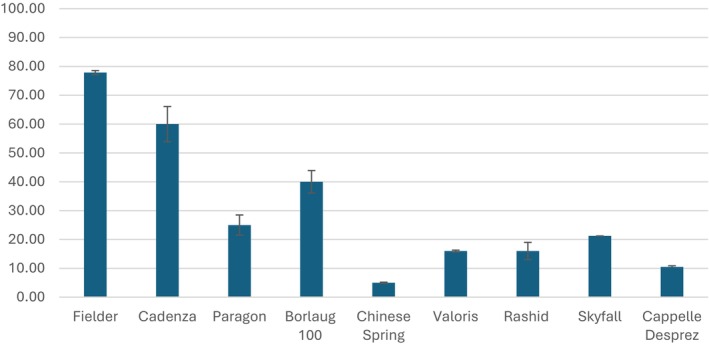
Transformation efficiencies in various wheat cultivars. Transformation efficiencies achieved using different GRF4‐GIF1 constructs across multiple wheat cultivars, including elite and commercial lines.

Transformation efficiencies with this construct reached 77.5% in Fielder, 60% in Cadenza, and 40% in Borlaug 100. In winter, wheat cultivars such as Valoris and Skyfall, efficiencies were 16 and 20%, respectively (Figure 2). Notably, multiple transgenic plants were often regenerated from a single embryo, and approximately 80% of these plants exhibited a copy number difference greater than three compared with each other (73 out of 91 plants). This indicates that the plantlets likely arose from independent transformation events. This suggests that the actual transformation efficiency may be even higher than reported. When the calculated on the basis independent regenerants per embryo, transformation efficiency in Fielder reached 98.3%. Consistent with the findings of Johnson et al. (2023), who reported that 84% of transgenic plantlets regenerated from the same embryos originated from independent transformation events. Only Fielder and Cadenza were amenable to transformation without the *GRF4‐GIF1* fusion, albeit with substantially lower efficiencies, 25 and 5% respectively.

This advancement has enabled successful transformation across a broader range of wheat genotypes, including elite and commercial cultivars, such as Borlaug 100, RGT Rashid, Skyfall, and Valoris. Among these, Skyfall is the most widely cultivated hexaploid bread making wheat in the UK, Valoris is a French variety notable for its high pentosan content, and Borlaug 100, developed by CIMMYT, serves as a key international breeding line (Table 1). Across all tested wheat cultivars, *GRF4‐GIF1* constructs consistently enabled successful transformation, although efficiency varied depending on the genotype and construct used. Skyfall, RGT Rashid, Valoris, and Cappelle Desprez are winter wheat varieties, and as such, are traditionally considered more recalcitrant to transformation than spring varieties; these varieties all showed lower transformation efficiencies (Figure 2). Given its consistent ability to enhance regeneration across diverse cultivars that could not be transformed without *GRF4–GIF1*, it can serve as a robust positive selection tool in wheat transformation experiments.

The growth of donor material is one of the main bottlenecks in wheat transformation. Donor materials are grown in controlled environment rooms (CERs). When plants are not healthy, transformation efficiency decreases across all cultivars. The *GRF4–GIF1* system enables transformation of all tested wheat cultivars regardless of factors, such as donor material quality, plant health, or the developmental stage of immature embryos. Additionally, it shortens the overall transformation timeline around 2 weeks as reported by Debernardi et al. (2020).

We observed high transgene copy numbers (greater than 10), which were associated with the phenotypic effects such as reduced seed number (Figure S1) when *GRF4‐GIF1* was driven by the *ZmUbi* promoter; any escapes were observed scarcely. Accordingly, we reduced the hygromycin concentration to promote reduced transgene copy number and lowered zeatin levels when *GRF4‐GIF1* was included in the construct (Table 2).

**Table 2:** Selection and regeneration conditions for *in vitro* wheat calli with or without GRF4‐GIF1 in different wheat cultivars

Wheat cultivar	GRF4‐GIF1	S1 Hygromycin (mg/L)	S2 Hygromycin (mg/L)	Regeneration hygromycin (mg/L)	Zeatin (mg/L)
Fielder	−	20	30	20	2.5
Fielder	+	15	15	15	1.0
Kronos	−	15	15	15	2.5
Kronos	+	15	15	15	2.5
Cadenza	−	10	10	10	1.0
Cadenza	+	10	10	10	1.0
Skyfall	+	10	10	10	2.5
Reedling	+	10	10	10	1.0
Cappelle Desprez	+	10	10	10	1.0
Paragon	+	5	5	5	1.0
Valoris	+	5	5	5	1.0
Chinese Spring	+	5	5	5	2.5

S1 and S2: Sequential selection media with increasing hygromycin concentrations. Regeneration Hygromycin: Hygromycin concentration during shoot regeneration. Zeatin: Concentration of zeatin in WRM during shoot regeneration. +/– GRF4‐GIF1: Indicates presence or absence of the GRF4‐GIF1 construct during co‐transformation.

### Effects on fertility and spike morphology

Analysis of mature plants revealed that GRF4–GIF1 insertion significantly reduced fertility across all cultivars (*P* < 0.001) (Figure S1). Lines with higher transgene copy numbers exhibited slightly greater sterility, with severity varying by cultivar. Among the cultivars tested, Kronos exhibited the lowest seed number, indicating the highest sensitivity to GRF4–GIF1, while Paragon and Cadenza were less affected.

Rudimentary basal spikelets (RBS) were more frequent in Cadenza and Skyfall lines carrying *GRF4–GIF1*, whereas other cultivars were largely unaffected. Total spikelet number and total spike number were significantly reduced across genotypes (*P* < 0.001) (Figure S2).

### Effects on yield‐related traits

The effect of *GRF4–GIF1* on thousand‐grain weight (TGW) was genotype‐dependent. Kronos experienced a negative impact, Chinese Spring showed a slight reduction, and Fielder displayed a positive effect. Across all cultivars, average grain area was slightly increased (*P* < 0.001) (Figure S2), consistent with previous observations (Debernardi et al., 2020).

### Effects on tiller number and plant architecture

*GRF4–GIF1* significantly increased tiller number (10 versus 15) in Fielder (*P* = 0.0014), with other genotypes showing similar, though non‐significant, trends. The number of spikes per plant (10 versus 15) was significantly increased in Fielder (*P* = 0.0015). Conversely, plant height was negatively affected across all genotypes (*P* < 0.001), with the magnitude of reduction varying among cultivars (Figure S2).

*GRF4–GIF1* expression induces a trade‐off between reproductive and vegetative traits. While spikelet fertility and total spikelet number are reduced, certain cultivars, such as Fielder, Cadenza, Paragon, and Reedling exhibit increased grain size, tiller number, and spike number, indicating cultivar‐specific compensatory effects (Figure S2). These findings underscore the critical role of promoter selection and genetic background in balancing high transformation efficiency with phenotypic stability.

In gene editing applications, segregation of the T‐DNA in subsequent generations typically eliminates these pleiotropic effects, enabling recovery of normal, edited lines. For transformation experiments targeting sensitive developmental traits, careful control of transgene expression is necessary to minimise unintended phenotypic consequences.

### Extending GRF4‐GIF1 to tetraploid wheat using different promoters

Based on our initial observations indicating increased sensitivity of the tetraploid wheat cultivar Kronos to *ZmUbi*‐driven *GRF4‐GIF1* overexpression, manifested by partial sterility and recognising that *ZmUbi* is a strong constitutive promoter, we developed a series of constructs designed either to modulate *GRF4‐GIF1* expression using alternative promoters or to facilitate its excision following transformation (Table 3). These constructs allowed us to investigate strategies for minimising phenotypic changes while maintaining high transformation efficiency, thereby tailoring the system to the specific requirements of tetraploid wheat.

**Table 3:** Constructs used in different tetraploid wheat cultivars

Construct name	Promoter driving GRF4‐GIF1	Reason for choosing	Marker gene	Addgene number
pGGG ZmUbi GRF4‐GIF1	ZmUbi::*TaGRF4‐GIF1*	Strong promoter	OsUbi::*GUS*	# 246478
pGGG OsAct GRF4‐GIF1	OsAct::*TaGRF4‐GIF1*	Medium promoter	OsUbi::*GUS*	# 246479
pGGG 35S GRF4‐GIF1	CaMV35S::*TaGRF4‐GIF1*	Weak promoter	OsUbi::*GUS*	# 246480
pGGG HSCre GRF4‐GIF1	Zm*Ubi*::T*aGRF4‐GRF1*	Excisable via Heat Shock	OsUbi::*GUS*	# 246481
pGGG PLTP GRF4‐GIF1	Zm*PLTP*::Ta*GRF4‐GIF1*	Tissue‐specific promoter	OsUbi::*GUS*	# 246482
pGGG GRF5‐GIF1	Zm*Ubi*::Ta*GRF5‐GIF1*	Strong promoter	OsUbi::G*US*	# 246483
pGGG GUS	Non‐GRF4‐GIF1 Control	Control	OsUbi::*GUS*	# 165418
pSoup HSCre GRF4‐GIF1	Zm*Ubi*::T*aGRF4‐GRF1*	Excisable via Heat Shock	N/A	# 246484

To broaden the scope of our analysis beyond Kronos, for which a TILLING mutant population is available, we evaluated these constructs in several additional tetraploid wheat cultivars, including CIRNO‐C (developed by CIMMYT), Om Rabi 5 (from ICARDA), and Svevo, an Italian durum wheat cultivar with full genomic sequence available.

Transformation efficiencies varied markedly among the tetraploid wheat cultivars tested. The Italian durum wheat variety *Svevo* exhibited the highest efficiency (55%) when transformed with the *ZmUbi::GRF4‐GIF1* construct. Kronos, CIRNO‐C, and Om Rabi showed lower efficiencies of 50, 11, and 4%, respectively. In the absence of *GRF4‐GIF1*, only Kronos and Svevo could be transformed, and at much lower frequencies (2 and 15%, respectively).

The *OsAct::TaGRF4‐GIF1* construct produced a more moderate effect (approximately 4%) but still performed better than the weak *CaMV35S* promoter and than the excisable and tissue‐specific promoter systems in both Kronos and CIRNO‐C. In contrast, Om Rabi and Svevo were successfully transformed using tissue‐specific and excisable promoters, reaching efficiencies of 4 and 2% in Om Rabi, and 15 and 19% in Svevo, respectively.

Overall, transformation outcomes depended on both the cultivar and the construct used. Notably, the highest efficiencies across all tetraploid lines were consistently obtained when the *GRF4‐GIF1* fusion was driven by the strong ZmUbi promoter, highlighting the importance of promoter strength in optimising *GRF4‐GIF1*‐mediated transformation.

### Zeatin concentration and regeneration

In many plant transformation systems, cytokinins are essential for shoot regeneration, with zeatin being the preferred cytokinin in wheat transformation. Previous high‐efficiency protocols have typically used zeatin at 5 mg L^−1^ (Ishida et al., 2015). In our study, Svevo, which exhibited greater regeneration capacity among all tested tetraploid wheat cultivars, achieved a transformation efficiency of 55% using the *GRF4–GIF1* construct with only 1.0 mg L^−1^ zeatin, indicating that the *GRF4–GIF1* fusion can promote shoot initiation and/or regeneration without the need for high zeatin concentrations. This finding aligns with Debernardi et al. (2020), who also reported high transformation efficiencies in Fielder without the addition of exogenous cytokinin. Svevo maintained a moderate transformation efficiency of 15% when transformed with the control construct that does not contain *GRF4–GIF1*, while Kronos showed 8.5% efficiency under the same conditions. In contrast, cultivars such as CIRNO‐C and Kronos demonstrated improved transformation outcomes when cultured with a higher zeatin concentration of 2.5 mg L^−1^, with Kronos reaching 50% efficiency. Notably, CIRNO‐C could not be transformed without the *GRF4–GIF1* construct; = All cultivars produced more plantlets per immature embryo when cultured with 2.5 mg L^−1^ zeatin (11–21 plantlets/embryo) compared with lower zeatin levels (1–17 plantlets/embryo). In particular, CIRNO‐C (21 plantlets/embryo) and Om Rabi 5 (11 plantlets/embryo) benefited most from the higher zeatin concentration, as their transformation efficiencies were lower; the additional zeatin helped increase the number of transgenic plantlets. In contrast, Kronos (2%–50% transformation efficiency) and Svevo (3%–55% transformation efficiency) already exhibited high regeneration rates, so culturing them with 1.0 mg L^−1^ zeatin was sufficient to produce a large number of plantlets from each embryo.

These results highlight the genotype‐specific hormonal requirements and underscore the critical role of *GRF4‐GIF1* in enabling the transformation of otherwise recalcitrant tetraploid wheat lines.

### Further controlling *GRF4‐GIF1* expression to minimise phenotypic effects

To mitigate undesirable phenotypic effect described above, particularly sterility associated with high transgene copy numbers, we considered the regulatory role of microRNA miR396, which naturally downregulates *GRF* expression in mature tissues (Debernardi et al., 2020). This effect may be more pronounced in tetraploid wheat, which could have a lower miR396‐mediated regulatory capacity compared with hexaploid cultivars.

We hypothesise that a high copy of *GRF4–GIF1* gene can overwhelm the endogenous miR396 pool, resulting in excessive GRF activity in mature plants and contributing to pronounced phenotypic effects. Vandeputte et al. (2024) observed a similar phenomenon in maize, where male sterility correlated with higher copy number variation in *ZmGRF4–GIF1 and TaGRF4–GIF1* T_0_ transgenic plants.

To address this, we employed two complementary strategies. First, tissue‐specific expression using the non‐constitutive *ZmPLTP* promoter restricts *GRF4–GIF1* activity to embryos and callus during transformation, minimising expression in mature plants. This promoter has been successfully used in maize for similar purposes (Lowe et al., 2018). Second, we applied a heat‐inducible Cre/lox excision system to remove the *GRF4–GIF1* cassette post‐regeneration. This allows excision of *GRF4–GIF1* after plant regeneration, reducing expression during later developmental stages and thereby mitigating strong phenotypic effects. Similar Cre/lox‐based excision strategies with Z*mBbm* and *ZmWus2* have been reported in maize (Wang et al., 2020).

These strategies were tested in both the hexaploid cultivar Fielder and the tetraploid cultivar Kronos, demonstrating that controlled expression can maintain high transformation efficiency while substantially reducing deleterious phenotypes. The need for construct redesign in tetraploid wheat arises from the combined effects of promoter choice, copy number, and differences in miR396‐mediated regulation between hexaploid and tetraploid genotypes. By explicitly linking these factors, we provide a mechanistic rationale for the observed genotype‐specific phenotypic outcomes and for the modifications made to optimise the system across wheat ploidy levels.

### Tissue‐specific expression using the non‐constitutive *ZmPLTP* promoter driven *GRF4‐GIF1*

In Fielder, the *ZmUbi::GRF4‐GIF1* construct delivered the highest transformation efficiency at 62% calculated as 1 plant per embryo, and producing an average of 3.1 plantlets per embryo. By contrast, the *ZmPLTP GRF4‐GIF1* construct achieved a transformation efficiency of 20%, generating 1.7 plantlets per embryo with a low escape rate of 0.4%. The *ZmUbi*::*GRF4–GIF1* construct resulted in a 33.6% transformation efficiency, whereas *ZmPLTP*::*GRF4–GIF1* yielded a 23% efficiency and was associated with improved seed number (Table 4). Kronos was most sensitive to the *ZmUbi*::*GRF4–GIF1* construct, which resulted in 46% sterility. In contrast, expression of *GRF4–GIF1* under the PLTP promoter caused only a moderate reduction in main seed number, an increase in spike number, and tiller number while TGW remained comparable to the control (57.87 g vs. 56.22 g in non‐transgenic plants), while maintaining fertility in approximately 85% of the transgenic plants, highlighting its suitability for minimising phenotypic disruption in sensitive genotypes. Also in Fielder, main seed number and TGW were improved.

**Table 4:** Phenotypic traits of Fielder and Kronos at maturity, including plant height, tiller number, spike number, thousand‐grain weight (TGW), and grain length, width, and area across different constructs

Variety	Construct	Main seeds ± SE	Weight(g) ± SE	TGW(g) ± SE	ØArea ± SE	ØWidth ± SE	ØLength ± SE	Tillers ± SE	Height (cm) ± SE	Spike number ± SE
Fielder	Control	448.24 ± 25.5a	21.44 ± 1.2a	48.48 ± 0.7b	16.25 ± 0.2b	3.44 ± 0.02b	6.49 ± 0.06c	14.67 ± 0.8b	85.52 ± 1.1a	14.62 ± 0.8b
	HS Cre	324.61 ± 19.2b	16.05 ± 0.8b	50.54 ± 0.9b	17.16 ± 0.3b	3.49 ± 0.03b	6.75 ± 0.05b	16.44 ± 0.7ab	81.42 ± 1.2a	16.12 ± 0.7ab
	Wox5	231.79 ± 27.9c	14.11 ± 1.5b	63.00 ± 2.0a	20.44 ± 0.5a	3.90 ± 0.06a	7.09 ± 0.07a	11.26 ± 0.7c	84.84 ± 2.4a	11.00 ± 0.7c
	PLTP	369.13 ± 18.4ab	17.28 ± 0.7b	48.25 ± 1.0b	16.41 ± 0.3b	3.42 ± 0.03b	6.62 ± 0.06bc	18.50 ± 0.6a	83.19 ± 1.0a	18.22 ± 0.6a
Kronos	Control	157.00 ± 11.7a	8.70 ± 0.6a	56.22 ± 1.4a	19.59 ± 0.3a	3.59 ± 0.04b	7.70 ± 0.05a	15.33 ± 0.9b	66.88 ± 0.9a	15.04 ± 0.9bc
	HS Cre	45.50 ± 13.0b	2.48 ± 0.7b	54.87 ± 1.1a	20.41 ± 0.5a	3.66 ± 0.04ab	7.85 ± 0.11a	15.10 ± 11.15b	61.25 ± 1.34bc	14.70 ± 1.3c
	Wox5	24.23 ± 6.2b	1.41 ± 0.4b	52.73 ± 2.4a	20.77 ± 0.6a	3.76 ± 0.05a	7.83 ± 0.13a	11.92 ± 1.1b	60.15 ± 1.78c	10.31 ± 1.1d
	PLTP	64.05 ± 14.8b	3.88 ± 0.9b	57.87 ± 1.2a	20.19 ± 0.3a	3.61 ± 0.03ab	7.80 ± 0.08a	23.95 ± 1.3a	65.15 ± 1.0ab	23.75 ± 1.3a

Values represent means ± SE. Different letters within the same column indicate significant differences according to Tukey's test at *P* ≤ 0.05.

Grain size parameters remained largely unchanged, indicating that PLTP‐driven expression supports phenotypic stability while enhancing transformation efficiency. This highlights PLTP as a suitable promoter for minimising adverse developmental effects in both Fielder and Kronos.

**Figure 3:**
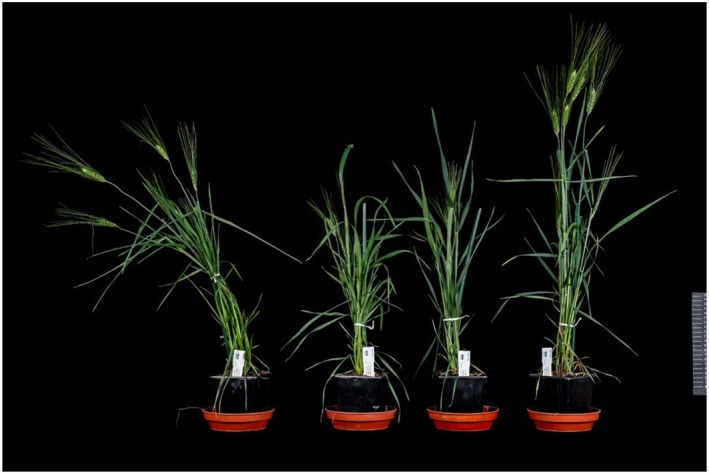
Phenotype of Kronos PLTP GRF4‐GIF1 and non‐transformed control plants (right). Transgenic Kronos plants expressing the ZmPLTP GRF4‐GIF1 construct showed phenotypes closely resembling non‐transformed controls, indicating minimal pleiotropic effects from transgene expression.

These results demonstrate that the promoters driving *GRF4–GIF1* expression strongly influence transformation efficiency. While stronger promoters enhance transformation efficiency, they also result in more undesirable phenotypic effects (Figure 3). In contrast, tissue‐specific expression driven by the *ZmPLTP* promoter enables efficient transformation while minimising associated fertility problems.

### Excision of *GRF4‐GIF1* via heat‐induced Cre‐lox system

Continued expression of morphogenic genes can interfere with downstream functional analysis and characterisation of trait genes. HS Cre‐Lox excision system can reduce the number of morphogenic genes inserted and improve plant development and fertility.

Using the HS Cre‐Lox system, transformation efficiencies reached 30% in Fielder and 15% in Kronos. Successful *GRF4–GIF1* excision was achieved in 11 of 15 plants (73%) in Kronos, whereas excision in Fielder occurred in 8 of 20 plants (13%). In both Fielder and Kronos, the HS Cre‐Lox system reduced seed number and total seed weight compared with non‐transgenic controls (Table 4), with the effect more pronounced in Kronos than in Fielder; however, TGW remained stable, indicating the production of fewer but normally sized grains. Extending the heat shock to 24 h slightly improved TGW but did not restore seed number. Notably, fertility in Kronos was markedly increased to 87%. Overall, the HS Cre‐Lox system helped restore fertility in both cultivars, with a more pronounced effect in Kronos.

Plant height was slightly reduced in Kronos, while in Fielder it remained similar to the control. Spike number increased moderately in Fielder but showed minimal change in Kronos. These results suggest that the HS Cre‐Lox system can achieve transgene excision but may still induce moderate fertility effects, especially in sensitive genotypes.

These outcomes are consistent with excision‐based improvements previously reported in maize (Wang et al., 2020), potentially reducing the copy number of *ZmBBM* and *ZmWUS2* from the maize genome and thus eliminating the detrimental ectopic expression even in plants still retaining some morphogenic gene sequences (Aesaert et al., 2022; Johnson et al., 2023). This technology has been extended to wheat where a higher copy number of Z*mBbm* and *ZmWus2* resulted in developmental defects. When using the heat shock inducible *moCRE* gene (*Zm‐Hsp17.7*_*pro*_ *:moCRE*), all the transgenic plants were fertile, further supporting the utility of this system in mitigating *GRF4‐GIF1* phenotypic associated effects.

### Co‐transformation

We explored co‐transformation as a method to introduce both *GRF4‐GIF1* and other GOIs into wheat. By using a two‐*Agrobacterium* system with separate T‐DNAs for *GRF4‐GIF1* and the GOI, we achieved transformation efficiencies comparable to those obtained with the *ZmUbi GRF4‐GIF1* construct. This approach offers greater flexibility and the potential for segregation of *GRF4‐GIF1* and GOI in subsequent generations.

We applied this co‐transformation strategy to several elite wheat cultivars, including Borlaug 100, Skyfall, Kronos, and Cadenza. These cultivars could not be transformed or showed very low transformation efficiencies when *GRF4‐GIF1* was not included. However, when constructs containing both the GOI with a selectable marker and a separate GRF4‐GIF1 expression cassette were introduced, regeneration efficiency increased markedly, resulting in a substantial improvement in overall transformation efficiency.

This strategy was applied to several elite wheat cultivars (Borlaug 100, Skyfall, Kronos, and Cadenza) that were previously recalcitrant or showed very low transformation efficiency. Inclusion of *GRF4‐GIF1* significantly increased regeneration efficiency and overall transformation success, similar in concept to the “altruistic transformation” system described in maize, where transient expression of Wus2 promotes somatic embryo formation while the selectable marker T‐DNA integrates stably (Hoerster et al., 2020).

### Comparison with other morphogenic gene

We also tested the wheat homologue of Arabidopsis WUSCHEL‐RELATED HOMEOBOX 5 (WOX5) based on previous reports indicating that WOX5 can enhance wheat transformation efficiency and reduce genotype dependence (Wang et al., 2022). *WOX5* was evaluated in both Fielder and Kronos cultivars. Selection of transformed plants was performed using the GOI‐linked selectable marker, and phenotypic effects were systematically recorded in mature T_0_ plants, including fertility (seed set), plant height, tiller number, spike number, and grain traits (area, length, and weight).

**Figure 4:**
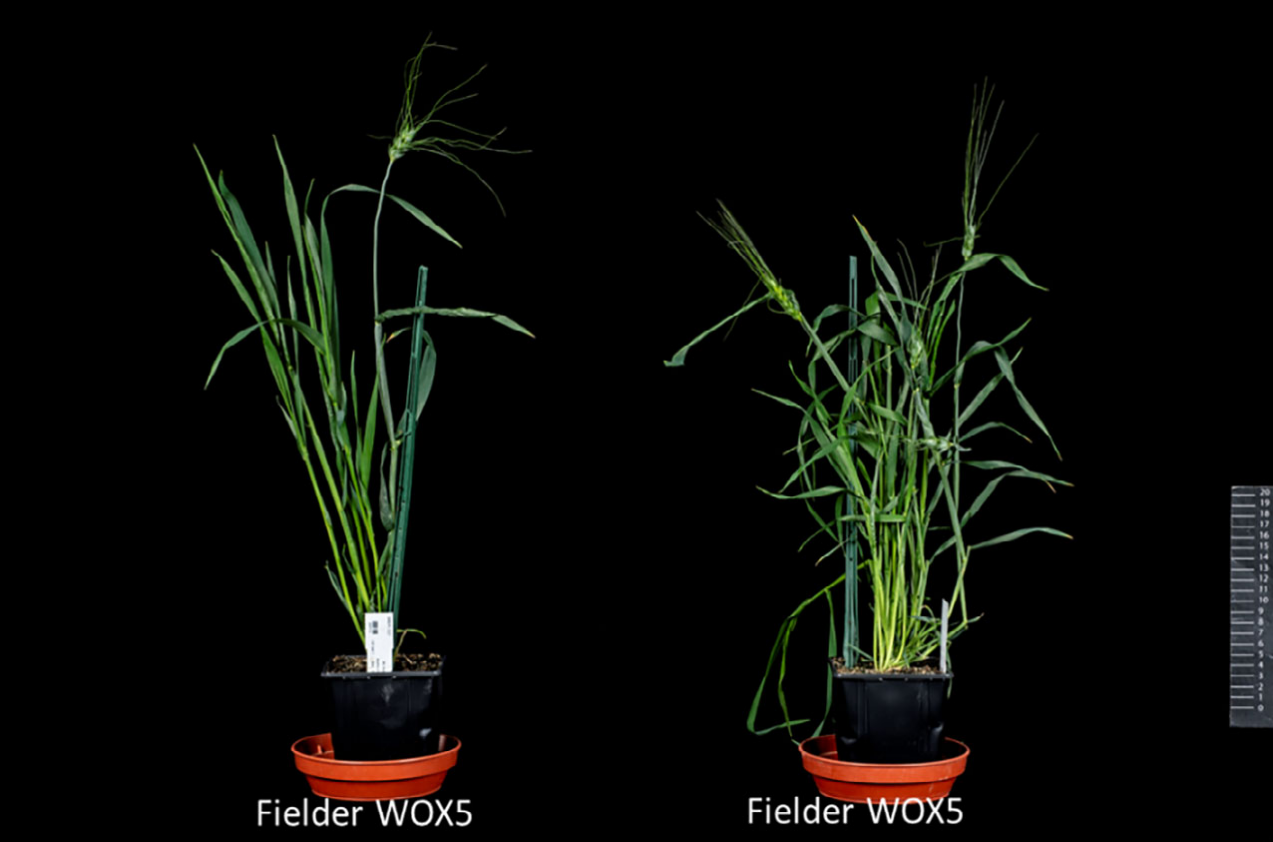
Phenotype of Fielder plants transformed with Wox5. Fielder plants expressing Wox5 exhibited distinct morphological changes compared with non‐transformed controls. The mature plants displayed a shorter stature, larger grain size, and wider, spiralled leaf shapes, indicating significant developmental effects associated with Wox5 overexpression.

Fielder, *ZmUbi::WOX5* yielded ~15% transformation efficiency, producing an average of 1.04 plantlets per line. In Kronos, transformation efficiency was slightly higher with *GRF4‐GIF1* constructs (~24%), but *WOX5* expression caused severe fertility penalties (~41% sterility) and morphological alterations, including wider, spiralled leaves (Figure 4). Grain area, weight, and length increased, whereas tiller and spike number decreased (Table 4).

In contrast, *GRF4‐GIF1* consistently produced higher transformation efficiencies with morphologically normal plants. In Kronos, PLTP‐ and HS Cre‐Lox‐driven *GRF4‐GIF1* lines restored fertility relative to ZmUbi‐driven *GRF4‐GIF1*, while WOX5 lines showed strong pleiotropic effects. This comparison highlights *GRF4‐GIF1* as a balanced morphogenic regulator, capable of enhancing regeneration without severe developmental penalties.

In summary, *GRF4‐GIF1* consistently achieved higher transformation efficiencies while producing morphologically normal plants, highlighting its advantage as a balanced morphogenic regulator for wheat transformation. In both Fielder and Kronos, all constructs reduced seed number relative to controls, but *WOX5* caused the strongest fertility penalty in Kronos, accompanied by a marked increase in TGW and grain size. In contrast, PLTP‐ and HS Cre‐Lox‐driven *GRF4‐GIF1* lines restored fertility in Kronos. While the HS Cre‐Lox system increased tiller and spike number, it did not fully restore overall yield. Among Kronos transgenic lines, *ZmUbi*‐driven *GRF4‐GIF1* exhibited reduced plant height and high sterility, whereas lines with HS Cre‐mediated excision or PLTP‐driven *GRF4‐GIF1* showed substantially recovered fertility. WOX5 expression primarily impaired fertility and produced pronounced morphological changes, including widened, spiralled leaves and thickened stems, despite increases in grain size.

## CONCLUSION

*GRF4–GIF1* enhances wheat transformation efficiency across diverse cultivars but induces a trade‐off between reproductive and vegetative traits, with high transgene copy numbers leading to reduced fertility, spikelet number, and seed set. These pleiotropic effects are genotype‐dependent, with sensitive cultivars such as Kronos being most affected, while others like Fielder can exhibit compensatory increases in tiller number, spike number, and grain size.

Promoter choice strongly influences the balance between transformation efficiency and phenotypic stability. Constitutive expression using *ZmUbi* drives high efficiency but exacerbates developmental defects, whereas tissue‐specific expression via the *ZmPLTP* promoter limits *GRF4–GIF1* activity to early developmental stages, maintaining high fertility and normal grain traits while still supporting efficient transformation. Optimisation of *GRF4–GIF1* expression, driven by promoters with differing strengths and temporal activities, in combination with varying levels of zeatin and hygromycin, further improves transformation outcomes across multiple tetraploid and hexaploid wheat cultivars.

Complementary strategies, such as the heat‐inducible Cre‐lox excision system, allow post‐regeneration removal of *GRF4–GIF1*, restoring fertility and mitigating phenotypic abnormalities, particularly in sensitive genotypes. Together, these approaches demonstrate that careful regulation of *GRF4–GIF1* expression through promoter selection, controlled excision, and media optimisation can optimise wheat transformation while minimising unintended developmental effects. These findings provide a framework for generating high‐efficiency, phenotypically stable transgenic and gene edited wheat lines.

## MATERIALS AND METHODS

### Plant material and growth conditions

In this study, we utilised selected hexaploid bread wheat (*Triticum aestivum* L.) cultivars, as well as a few tetraploid durum wheat (*Triticum durum* Desf.) cultivars (Table 1). Small batches of wheat seeds from different cultivars were sown at weekly intervals to ensure a continuous supply of immature embryos. Donor plants were grown under controlled environmental conditions at 18 ± 1°C (day) and 15 ± 1°C (night), with 65% relative humidity and light levels of 600 μmol m^−2^ s^−1^ provided by a combination of fluorescent and tungsten lighting in controlled environment growth rooms as described in Hayta et al., 2021. Winter wheat cultivars were exposed to a 4‐week vernalisation period at temperature 5°C, under a short‐day, 8 h light:16 h dark photoperiod, 70 μmol m^−2^ s^−1^.

### Construct design and preparation

The hypervirulent *Agrobacterium tumefaciens* strain AGL1 (Lazo et al., 1991) was used in all plant transformation experiments. All transformation vectors were based on the binary vector pGoldenGreenGate (pGGG) (Smedley et al., 2021) and were assembled using MoClo golden gate assembly (Weber et al., 2011). Each pGGG vector contained the hygromycin selectable marker (hpt) and Cat1 intron driven by the switchgrass ubiquitin promoter (PvUbi) (Addgene # 246468), the *GRF4‐GIF1* gene fusion driven by various promoters or excisable, and the β‐glucuronidase gene (*GUS*) with two introns (*GUS*2Int) under the control of the rice ubiquitin promoter (Addgene # 246471). The pGGG vectors were electroporated into the *Agrobacterium* AGL1 competent cells with the helper plasmid pAL155 (Addgene # 246472), which contains an additional *VirG* gene as previously described in Hayta et al. (2019).

Single colonies of AGL1 containing the desired constructs were inoculated into 10 mL LB medium supplemented with appropriate antibiotics and incubated at 28°C, shaking at 200 rpm for approximately 65 h. A modified version of the Tingay et al. (1997) protocol was used to prepare standard *Agrobacterium* inocula, following Bartlett et al. (2008). Equal volumes of sterile 30% glycerol and bacterial culture were mixed and aliquoted (400 μL) into 0.5 mL tubes. These inocula were stored at −80°C until use.

The *GRF4‐GIF1* gene fusion was synthesised as a MoClo golden gate level 0 component, internal *Eco*31I and *Bpi*I restriction enzyme sites were removed by silent mutation and standardised MoClo overhangs and *Eco*31I sites added for assembly. The level 0 *GRF4‐GIF1* CDS was assembled into expression cassettes in the Level 1 Position 2 pICH47742 (Addgene # 48001) with the promoters listed in Table 3 and the Nos terminator. All the regular promoters listed were at our laboratory's disposal; however, the *Zea mays* phospholipid‐transfer protein (*ZmPLTP*) promoter was designed and synthesised as a MoClo golden gate Level 0 component, internal *Eco*31I and *Bpi*I restriction enzyme sites removed and standardised MoClo overhangs and *Eco*31I sites added for assembly. The pGGG GRF4‐GIF1 GUS (Addgene # 246478) vector containing the maize ubiquitin promoter (*ZmUbi*) driving the *GRF4‐GIF1* and the GUS marker gene served as a control.

The inducible site‐specific recombinase (Cre) recombinase CDS under the control of the barley heat‐shock inducible promoter 17 (*HvHsp17*) was deployed for conditional excision of the ZmUbi::GRF4‐GIF1::NosT and self‐excision of the *HvHsp17*::Cre::HSTerm. A loxP acceptor cassette was designed and synthesised (Genewiz), which enabled the insertion via *Bpi*I of Level 1 expression cassettes flanked by loxP sites. The cassette in turn could be isolated via *Eco*31L and cloned into any MoClo L1 acceptor plasmid. The L1P2 *HvHsp17*::CreInt::*HvHsp17*::Term and L1P1 *ZmUb*i::*GRF4‐GIF1*::Nos were assembled into the loxP cassette, and then the whole assembly was inserted into pICH47811 (AddGene #48008); this vector was deemed L1P2 Rev Cre/LoxP heat shock excisable GRF4‐GIF1 (Addgene # 246486), before cloning in the reverse orientation upstream of the *hpt* selection cassette in the final Level 2 pGGG binary vector.

Constructs with two separate T‐DNAs provide the opportunity to either use “old” constructs in elite wheat cultivars without having to re‐clone to insert *GRF4‐GIF1* or to obtain *GRF4‐GIF1*‐free plants through segregation in later generations. A T‐DNA cassette was designed and synthesised which was flanked by left and right border repeat sequences and was classically cloned into the pSoup helper plasmid (Addgene # 165419) via the *Kpn*I and *Hind*III restriction enzyme sites. The L1P2 Cre *lox*P GRF‐GIF Cassette (Addgene # 246486) was cloned into the pSoup's T‐DNA via *Eco*31L using a golden gate assembly. The resulting plasmid known as pSoup HSCre *GRF4‐GIF1* (Addgene # 246484) can be co‐electroporated into AGL1 with pGGG‐based plasmids or an *Agrobacterium* culture containing pSoup HSCre GRF4‐GIF1 can be mixed with an *Agrobacterium* culture containing a suitable plasmid of interest and co‐transformed into wheat.

Various *GRF4‐GIF1* constructs, driven by different promoters, were designed for testing in tetraploid wheat, which is more sensitive to phenotypic changes than hexaploid bread wheat (Table 3). Additionally, *GRF5‐GIF1* was included as an alternative construct to assess its potential in tetraploid wheat, alongside Ta*WOX5*, which was tested in Kronos and Fielder.

### *Agrobacterium‐*mediated transformation of different wheat cultivars

#### Immature embryo isolation and transformation procedure

Immature embryos were isolated from wheat grains harvested approximately 14 days post‐anthesis (early milk stage, GS73), when embryos measured 1–1.5 mm in diameter. Kernels from florets 1 and 2 of central spikelets were selected for transformation. Spikes were trimmed and surface‐sterilised with 70% (v/v) ethanol for 1 min, followed by 10% (v/v) sodium hypochlorite for 7 min, and rinsed three times with sterile distilled water.

Under aseptic conditions, immature embryos were excised using fine forceps under a stereomicroscope and placed in wheat inoculation medium (WIM) containing Murashige and Skoog (MS) salts, glucose, MES buffer, Silwet L‐77, and 100 μM acetosyringone (AS).

#### *Agrobacterium* preparation and co‐cultivation

*A. tumefaciens* cultures were prepared 1 day prior to transformation and grown overnight in MG/L medium at 28°C, shaken at 200 rpm. Bacterial cells were pelleted and resuspended in WIM to an optical density of OD_600_ = 0.5, supplemented with 100 μM AS, and incubated for 4–6 h at room temperature before inoculation.

Immature embryos were inoculated with the *Agrobacterium* suspension and co‐cultivated on solidified WIM containing AgNO_3_, CuSO_4_, and agarose at 24 ± 1°C in darkness for 3 days.

#### Callus induction and selection

After co‐cultivation, embryogenic axes were excised and transferred to wheat callus induction medium (WCI) containing 2 mg L^−1^ Picloram, 0.5 mg L^−1^ 2,4‐D, and Timentin. Calli were sequentially selected on media containing two levels of hygromycin: Selection 1 (S1) and Selection 2 (S2), each for 2 weeks. The specific hygromycin concentrations used at each selection stage varied depending on the wheat cultivar (Table 2).

#### Regeneration and rooting

After the first week of S2, embryogenic calli were transferred to light conditions (100 μmol m^−2^ s^−1^, 16 h photoperiod) to initiate shoot regeneration. Regeneration was performed on wheat regeneration medium (WRM) containing MS salts, sucrose, MES buffer, Timentin, and cultivar‐specific concentrations of hygromycin and zeatin riboside (Table 2), at 24 ± 1°C under a 16 h photoperiod. Shoots (1–2 cm) exhibiting root primordia were subsequently transferred to hormone‐free MS medium to promote root development.

#### Plant acclimatisation

Fully rooted plantlets were gently washed and transferred to soil in cell trays covered with propagator lids for acclimatisation. Plants were maintained under controlled environmental conditions (18 ± 1°C day / 15 ± 1°C night, 65% relative humidity, 400–600 μmol m^−2^ s^−1^ light intensity).

#### Co‐transformation system

To evaluate co‐transformation efficiency, *Agrobacterium* containing the GOI construct was co‐inoculated with *Agrobacterium* harbouring the pSoup HSCre GRF4‐GIF1 construct. Overnight cultures were adjusted to OD_600_ = 0.4 and mixed at a 3:2 ratio of GOI:GRF4‐GIF1 prior to embryo inoculation. Alternatively, pGGG vectors lacking GRF4‐GIF1 were electroporated into *Agrobacterium* strains already containing pSoup HSCre GRF4‐GIF1, and inocula were prepared and used for transformation following the standard procedure described by Hayta et al. (2019, 2021).

#### Heat‐shock treatment for inducible site‐specific recombinase (Cre) to excise *GRF4‐GIF1*

Plant material transformed with constructs containing the heat‐shock inducible Cre/lox‐mediated excision system for *GRF4‐GIF1* was subjected to a 40°C heat treatment for 16 h (overnight) during the second‐regeneration phase. Only calli that had developed green shoots were exposed to heat‐shock prior to sub‐culturing onto fresh media for further development.

#### DNA extraction and copy number analysis

Leaf samples 0.5–0.7 cm were collected, and DNA extracted using the protocol adapted from (Pallotta et al., 2003). Transgene presence and copy number were determined by quantitative real‐time PCR (qPCR) using TaqMan assays targeting the hygromycin resistance gene (hpt) and the endogenous CO_2_ (Constans‐like, AF490469) gene as described by Hayta et al. (2019).

Primers and probes were designed using the “TaqMan Probe and Primer Design” module in Primer Express (Applied Biosystems). Reactions were performed using the Absolute qPCR Mix (Low ROX version; Catalogue No. AB1318B, ThermoScientific) in a multiplex format detecting both *hpt* and *CO2* targets simultaneously.

Each reaction contained 200 nM of each primer and probe, and 5 μL of DNA template adjusted to a final concentration between 1.25–10 ng μL^−1^ (6.25–50 ng total DNA per reaction). Amplifications were carried out on an Applied Biosystems QuantStudio 5 system.

Thermal cycling conditions were as follows: initial enzyme activation at 95°C for 15 min, followed by 40 cycles of denaturation at 95°C for 15 s and annealing/extension at 60°C for 60 s.

The values obtained were used to calculate transgene copy number using the 2^–ΔΔCT^ calculation as described in (Livak & Schmittgen, 2001).

### Transformation efficiency calculation

The transformation efficiency throughout this article was calculated as single events occurring from each embryogenic line (i.e., one regenerated plant derived from one embryo) and displayed as the percentage of positive transgenic plants produced from the total number of immature embryos isolated and inoculated with *Agrobacterium* in an experiment. Transgenesis was confirmed by qPCR.

### Data collection

At maturity, full phenotypic traits, including plant height, tiller number, spike number, TGW, and grain length, width, and area across were measured. Grain traits were quantified using a MARVIN Seed Analyser (GTA Sensorik GmbH; hereafter referred to as “MARVIN”).

### Statistical analysis

Data were analysed using ANOVA on phenotypic data in RStudio (v1.3.1056). Means were compared using Tukey's HSD test with a significance level of *P* < 0.05.

## Supporting information


**Figure S1.** Somatic embryos produced in Fielder and Kronos on R1 medium.

**Figure S2.** In wheat varieties transformed with the ZmUbi::GRF4‐GIF1 construct, (a) spike number, tiller number, and average grain area increased, whereas plant height, spikelet number, and spike fertility were reduced. Changes in thousand‐grain weight (TGW) depended on the cultivar. (b) Transgene copy number and seed number.

## Data Availability

The data that support the findings of this study are openly available in Addgene at https://www.addgene.org/JIC_Wheat_Transformation/.
