# Supplementary figures and images for: Controlling GRF4‐GIF1 expression for efficient, genotype‐independent transformation across wheat cultivars
Source: Plant J. 2026 Mar 17;125(6):e70799. doi: 10.1111/tpj.70799 (PMC12995506; doi:10.1111/tpj.70799)

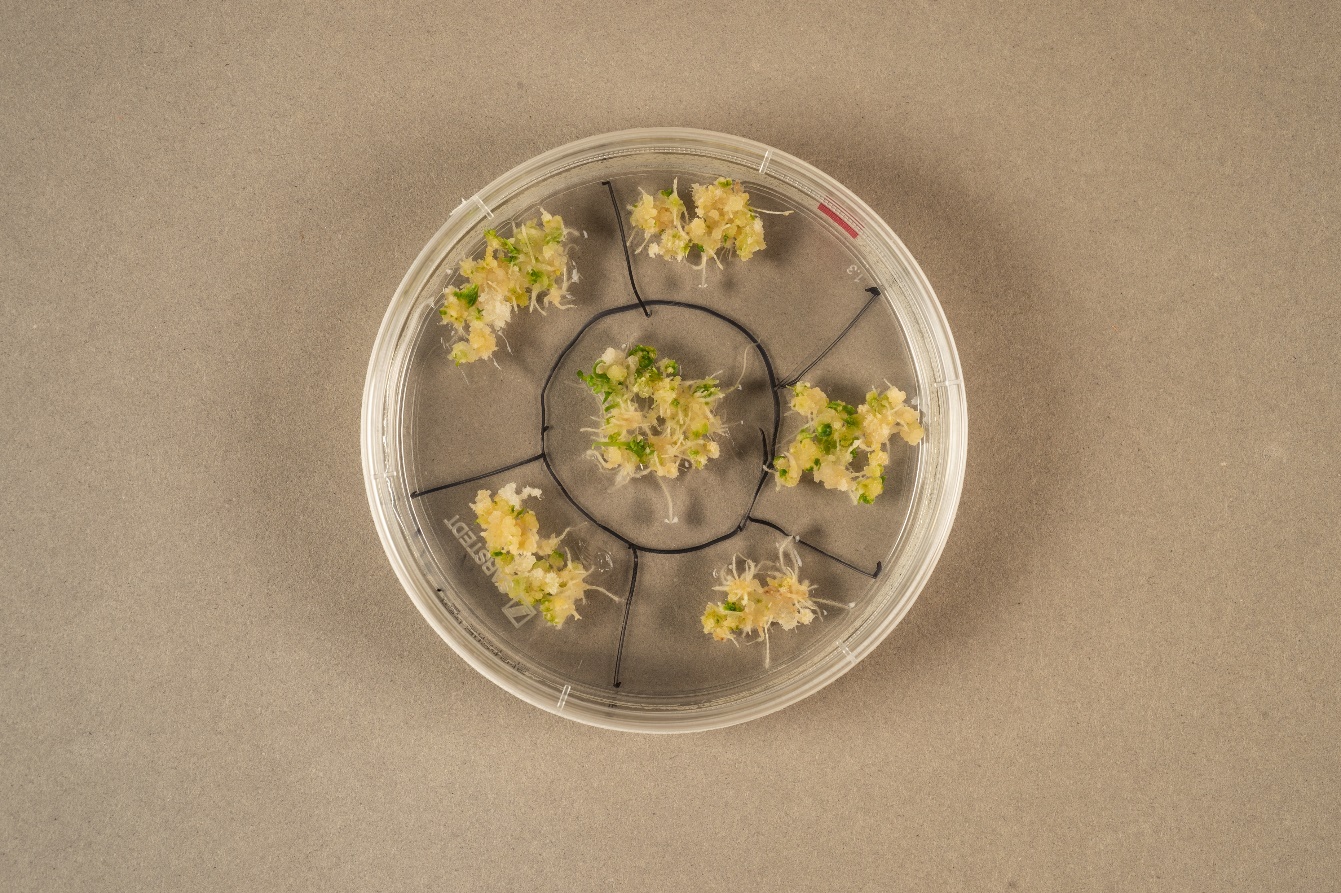

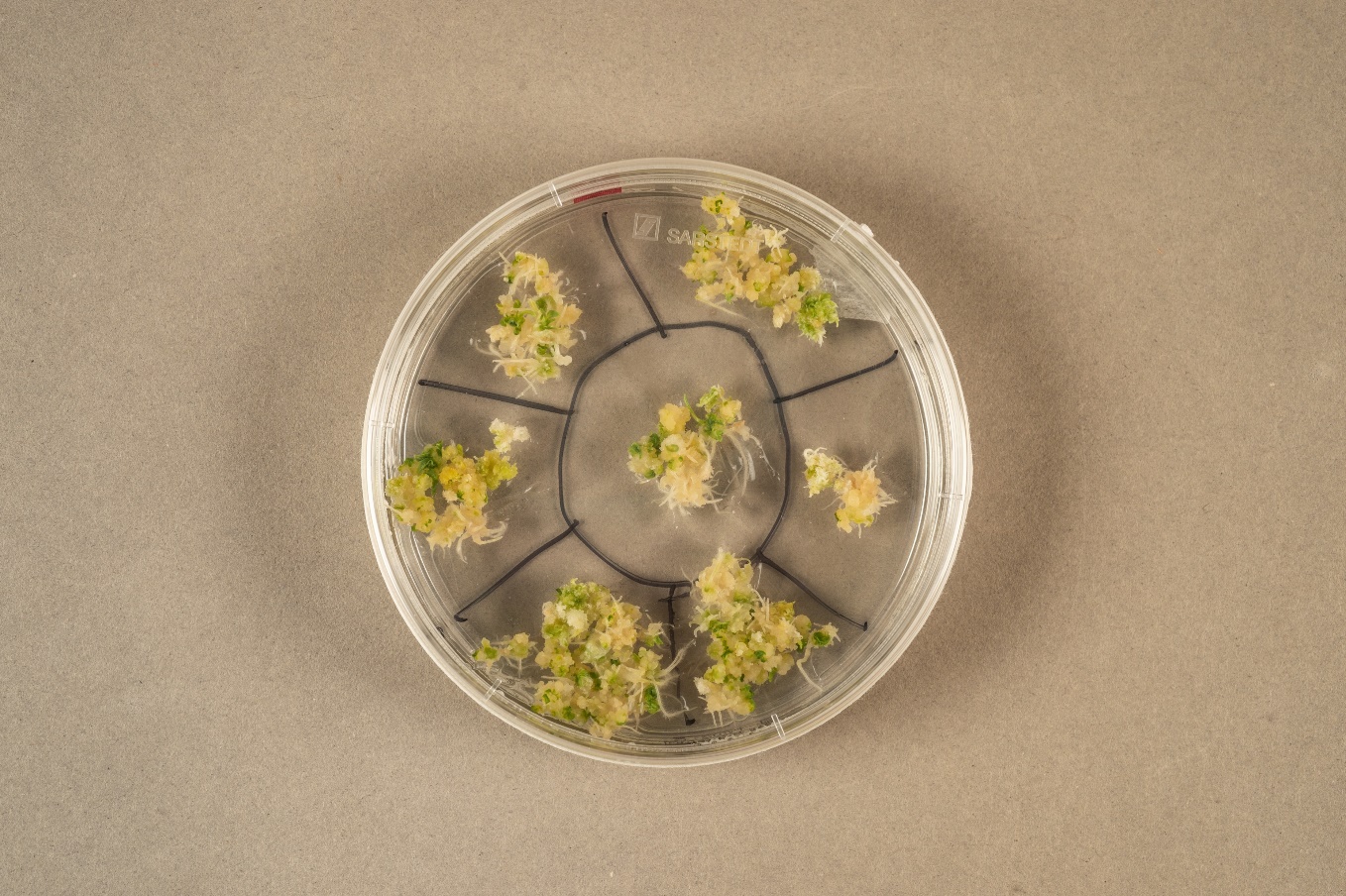


**Supplementary Fig. 1.** Somatic embryos produced in Fielder and Kronos on R1 medium.

Supplement: Supplementary file 1 — Figure S1. Somatic embryos produced in Fielder and Kronos on R1 medium. [file TPJ-125-0-s002.docx]
