# Supplementary material for: Controlling GRF4‐GIF1 expression for efficient, genotype‐independent transformation across wheat cultivars
Source: Plant J. 2026 Mar 17;125(6):e70799. doi: 10.1111/tpj.70799 (PMC12995506; doi:10.1111/tpj.70799)
Supplement: Supplementary file 2 — Figure S2. In wheat varieties transformed with the ZmUbi::GRF4‐GIF1 construct, (a) spike number, tiller number, and average grain area increased, whereas plant height, spikelet number, and spike fertility were reduced. Changes in thousand‐grain weight (TGW) depended on the cultivar. (b) Transgene copy number and seed number. [file TPJ-125-0-s001.docx]

**
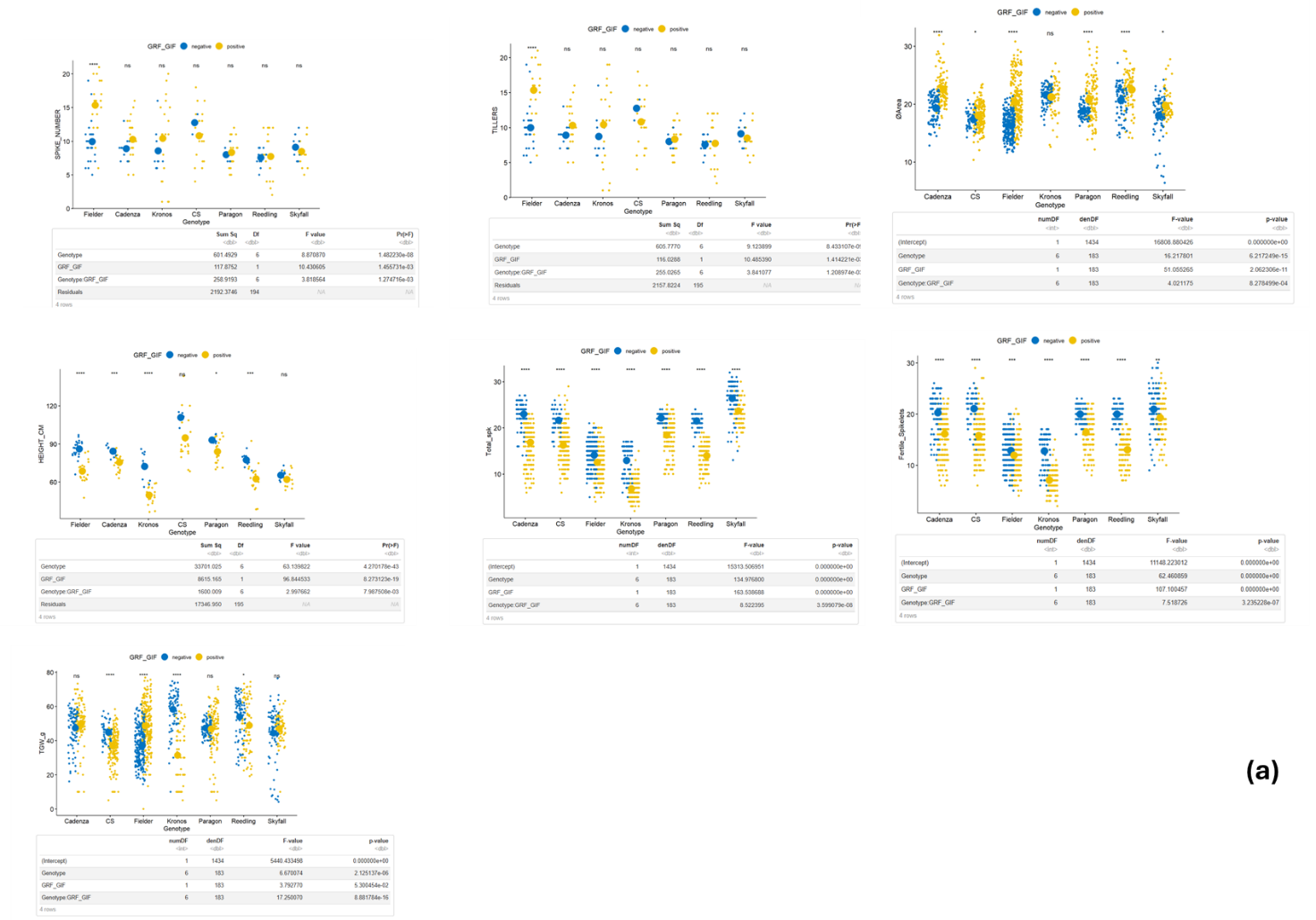
**

**
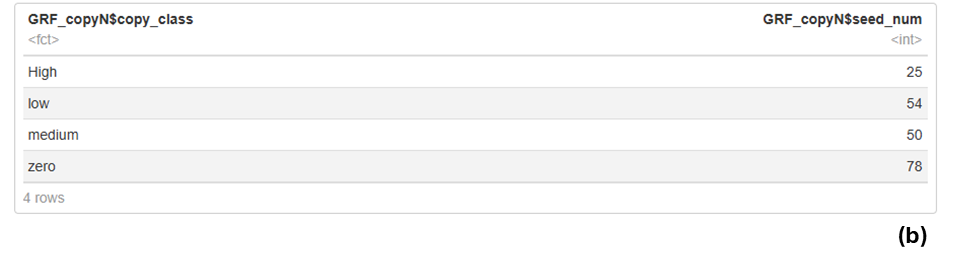
**

**Supplementary Figure 2.**
In wheat varieties transformed with the ZmUbi::GRF4-GIF1 construct, **(a)** spike number, tiller number, and average grain area increased, whereas plant height, spikelet number, and spike fertility were reduced. Changes in thousand-grain weight (TGW) depended on the cultivar. **(b)** Transgene copy number and seed number.
